# Effect of Rearing, Physiological, and Processing Conditions on the Volatile Profile of Atlantic Salmon (*Salmo salar*) Using SIFT-MS

**DOI:** 10.3390/foods14142540

**Published:** 2025-07-21

**Authors:** Manpreet Kaur, Konrad Dabrowski, Kevin J. Fisher, Md Zakir Hossain, Sheryl Barringer

**Affiliations:** 1 Department of Food Science and Technology, The Ohio State University, Columbus, OH 43210, USA; kaur.333@osu.edu (M.K.); hossain.154@osu.edu (M.Z.H.); 2 School of Environment and Natural Resources, The Ohio State University, Columbus, OH 43210, USA; fisher.645@osu.edu

**Keywords:** salmon, SIFT-MS, off-odors, quality, rearing, processing, physiological, lipid-oxidation, protein-degradation, geosmin, volatiles

## Abstract

This study examined the effects of rearing, physiological, and processing conditions on the volatile profile of Atlantic salmon. Fish were reared under two different temperature and light conditions, and three harvests were conducted at different time points for male and female fish. Fish were processed to yield fillets with or without skin. Volatiles were analyzed using SIFT-MS headspace analysis. Atlantic salmon reared in cooler temperatures under a 12 h light/dark cycle exhibited significantly lower concentrations of off-odor volatiles compared to those reared in warm conditions under continuous light, suggesting that cooler temperatures with a dark cycle help maintain freshness. A temperature shift from cool to warm further increased volatile accumulation. Longer rearing time resulted in higher volatile concentrations, attributed to greater biochemical products, increased susceptibility to lipid oxidation, protein degradation, and contaminant accumulation from the rearing environment. Males had higher volatile levels at 202 days, while females surpassed males by 242 days, likely due to increased biochemical accumulation associated with reproductive development. Fillets with skin exhibited significantly higher concentration of off-odor volatiles. These findings highlight the role of all studied factors in establishing optimum conditions to minimize spoilage-related volatiles and preserve the freshness of Atlantic salmon, with rearing temperature being the most critical factor.

## 1. Introduction

Atlantic salmon (*Salmo salar*) is a cornerstone of the United States seafood market, valued for its rich flavor, versatility across culinary applications, and exceptional nutritional profile, particularly its high omega-3 fatty acid content. Among these attributes, flavor remains a primary driver of consumer preference. The flavor of Atlantic salmon is closely linked to its volatile organic compounds (VOCs), which govern aroma, perceived freshness, and overall sensory quality [1,2,3]. These volatile organic compounds, including aldehydes, alcohols, and ketones, are created during biochemical processes such as lipid oxidation and protein degradation.

Lipid oxidation and proteolysis, accelerated by enzymatic activity and environmental stressors, generate off-odors and off-flavors such as rancidity and fishiness, diminish nutritional value, and ultimately shorten shelf life [4,5,6]. This deterioration not only reduces consumer acceptance but also contributes substantially to food waste and economic loss. Globally, it is estimated that 35% of seafood is discarded due to quality degradation [7]. As demand for Atlantic salmon continues to rise, the United States imported 105,001 metric tons of Atlantic salmon, valued at nearly $1.4 billion, during the first quarter of 2025. This represents a slight increase of less than 1% in volume and approximately 4% in value compared to the 104,606 metric tons, worth $1.3 billion, imported during the same period in 2024 [8]. Understanding and mitigating spoilage mechanisms is critical to sustaining market growth and minimizing environmental impacts associated with food waste.

The volatile profile of Atlantic salmon is not static, but a dynamic outcome shaped by a complex interaction of environmental factors. Rearing conditions, particularly water temperature and photoperiod (light–dark cycles), have significant influence on lipid metabolism, stress responses, and muscle composition. Elevated rearing temperatures, while promoting faster growth, may increase oxidative stress and the subsequent formation of aldehydes such as hexanal and nonanal during storage [9,10]. Similarly, photoperiod manipulation, commonly employed to regulate maturation, alters smoltification processes and muscle development, potentially affecting post mortem enzymatic activity and volatile organic compound generation [11,12]. Water quality parameters, including nutrient loading and microbial activity, further modulate Atlantic salmon flavor profiles. Nutrient-rich waters foster cyanobacterial blooms, elevating concentrations of compounds such as geosmin and 2-methylisoborneol, which are associated with undesirable, earthy off-flavors [13,14].

Beyond environmental influences, harvest time may also influence Atlantic salmon’s volatile profile. Atlantic salmon harvested during active growth phases typically retain higher glycogen reserves, influencing post mortem pH decline and the progression of rigor mortis. In contrast, delayed harvesting can accelerate proteolytic activity and microbial proliferation, both of which intensify the formation of spoilage-related volatile organic chemicals [15,16]. Biological factors such as sex may introduce variability in volatile organic compound production, given the distinct growth and maturation patterns between male and female Atlantic salmon [17].

Processing decisions, such as the choice to retain or remove the skin, also introduce additional complexity. The skin serves as a protective barrier against oxidative damage and microbial invasion but also harbors endogenous enzymes like lipases, proteases, and microbiota that can drive spoilage. Fillets with skin typically exhibit slower microbial colonization yet retain enzymatic activity that degrades lipids and proteins, contributing to volatile organic compound production [18,19,20]. Conversely, skin removal exposes muscle tissue to oxygen, accelerating lipid oxidation and protein degradation, promoting the formation of volatile organic compounds associated with off-odors [21,22]. Additionally, the lipid-rich epidermis of the skin can retain lipophilic compounds such as geosmin, potentially intensifying earthy notes in fillets with skin [23]. These observations emphasize the role of skin presence in volatile organic compound formation during storage.

Despite notable advancements in aquaculture and seafood preservation technologies, critical gaps remain in understanding how these diverse factors influence the volatile profile and shelf stability of farmed Atlantic salmon. Existing studies have primarily focused on growth performance and maturation, without systematically linking these biological and environmental variables to spoilage-related changes in volatile composition [9,10,11,12,13,14,15,16,17,18,19,20,21,22,23]. This approach limits the development of integrated strategies to optimize flavor stability and extend product shelf life.

This study addresses these gaps by investigating how rearing, physiological, and processing conditions including rearing temperature, light exposure, water flow systems, harvest timing, sex, and skin presence affect the volatile profile of Atlantic salmon fillets. The objective is to better understand the mechanisms driving the formation of spoilage-related volatiles under these conditions. We hypothesize that cooler rearing temperatures, a 12 h light/dark photoperiod with a recirculating aquaculture system, timely harvest based on age and sex, and skin removal before frozen storage will reduce spoilage-related volatiles and result in fresher salmon fillets. By identifying the specific factors that influence freshness and off-odor development, this research provides insights to help producers regulate rearing practices, refine harvest schedules, and optimize post-harvest handling. Ultimately, these findings aim to enhance product consistency, reduce waste, and support the delivery of high-quality salmon that meets consumer expectations.

## 2. Materials and Methods

### 2.1. Experimental Design and Rearing Conditions

**Figure 1:**
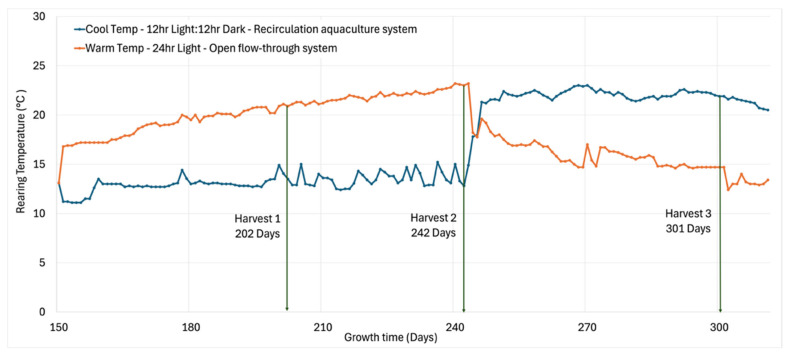
Rearing conditions—temperature, light exposure and water circulation system—during growth time of Atlantic salmon.

Before the study began, all procedures were approved by the Ohio State University Institutional Animal Care and Use Committee (2008A0220-R5, 2008A021-R5). The date of approval was 14 April 2024. Atlantic salmon (*Salmo salar*) were reared in the Aquaculture Laboratory at the School of Environment and Natural Resources, The Ohio State University, using a recirculating aquaculture system (RAS) specifically designed to facilitate controlled variation in water temperature and light exposure. The RAS setup included multiple tanks connected through a mechanical filtration and sump pump system. The system allowed for periodic switching between fresh city water and recirculated chilled water via water cutoff valves. This design enabled separation and independent manipulation of environmental variables between two sets of tanks. Fish were reared in 400 L tanks, with stocking densities of 39 fish/tank on day 202, 36 fish/tank on day 242, and 33 fish/tank on day 301. Tank size and fish density were maintained to support normal growth and development across all sampling points. Two distinct rearing conditions were maintained to simulate variable aquaculture environments: cool temperature (13.1 ± 0.85 °C) with 12 h light–12 h dark cycle and warm temperature (20.3 ± 1.95 °C) with continuous light [24] (Figure 1). The warm conditions used an open flow-through system. After 242 days, fish reared in cool conditions were acclimated to warm conditions over 2–3 days, and those in warm conditions were acclimated to cool conditions. Water temperatures were modulated using a chiller–recirculation system, while lighting conditions were controlled through scheduled illumination. Fish were fed Extruded Salmon (Skretting, UT, USA) ad libitum throughout the trial to avoid nutritional limitations on growth or flavor development.

### 2.2. Harvest and Sample Preparation

Fish were harvested at different time points to assess growth stage effects on volatile profiles. The first harvest was performed at 202 days, second harvest at 242 days, and third at 301 days, which was 59 days after the temperatures were reversed. Six fish were harvested at each time point, consisting of three males and three females.

At each harvest point, fish were euthanized humanely by a blow to the head and immediately bled via dorsal artery severance. Each fish was weighed, length-measured, and filleted. The fillets were separated into two categories: fillet with skin (muscle with skin) and fillet without skin (muscle without skin). In fillet without skin, the skin was removed manually by pulling it off the muscle, ensuring the complete removal of the skin. For analysis, only the muscle portion was used. Therefore, subcutaneous fat and red muscle were likely minimal or largely excluded from the final homogenized samples. Samples were stored at −80 °C for 30 days for later analysis.

### 2.3. SIFT-MS Headspace Analysis of Volatile Compounds

A 2 g sample from the head side of each fillet (skin-on or skin-off) was finely minced and placed in a 500 mL Pyrex bottle, followed by the addition of 20 mL of 0.5% ethanol to facilitate volatile extraction. The bottle was sealed using an open-top, septum-lined cap and homogenized using vortex mixer for 1 min. Samples were equilibrated in a shaking water bath at 42 °C for 30 min to allow headspace volatiles to stabilize. Volatile compounds were analyzed using selected-ion flow-tube mass spectrometry (SIFT-MS) (Voice200ultra, Syft Technologies, Christchurch, New Zealand). The volatiles compounds analyzed in this study are listed in Table 1. Analyses were conducted in selected-ion mode (SIM), employing precursor ions H_3_O^+^, NO^+^, and O_2_^+^. Quantification of volatile compounds was performed using known reaction rate coefficients for ion–molecule reactions. Calibration of the instrument was conducted using a certified gas standard containing benzene, ethylbenzene, toluene, and xylene isomers. The instrument’s response was validated against known concentrations to ensure accuracy prior to sample analysis. During the test, a 14-gauge passivated needle was used to pierce the septum for sampling, with the inlet temperature maintained at 175 °C. Each sample was analyzed over a 120 s run. Six replicates were analyzed per sample type. Background levels were determined using an empty Pyrex bottle as blank.

### 2.4. Statistical Analysis

All statistical analyses were conducted using JMP^®^ Pro Version 16.0.0 (Statistical Discovery, Cary, NC, USA). All graphical representations were generated using MATLAB^®^ R2024b Update 5 Version 24.2.0.2863752 (Mathworks, Natick, MA, USA). A two-way Analysis of Variance (ANOVA) was performed to examine the main effects. The two factors were rearing conditions (temperature, light, water circulation system) × harvest time; sex (male/female) × harvest time; and skin presence (skin-on/skin-off) × harvest time. Fisher’s Least Significant Difference (LSD) test was used for post hoc comparisons. Statistical significance was established at *p* ≤ 0.05. For each factor tested, three biological replicates were included, with a total of six fish sampled per harvest.

## 3. Results and Discussion

### 3.1. Effect of Rearing Temperature and Light on the Volatile Profile of Atlantic Salmon

**Figure 2:**
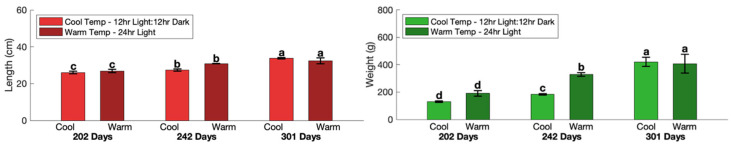
Effect of rearing temperature, light and harvest time on weight and length of Atlantic salmon. Different letters indicate significant difference in each factor (*p* < 0.05).

The rearing temperature and light conditions had a significant effect on the growth of Atlantic salmon, including on their total body weight and length (Figure 2). Fish reared under warm temperatures with continuous light showed higher growth rates, as indicated by a significantly greater total body weight and length, compared to those reared in cooler temperatures with a 12 h light–12 h dark cycle. The effect of harvest time on growth is also evident, with fish harvested at 242 days consistently showing significantly greater total body weight and length than those harvested at 204 days.

**Figure 3:**
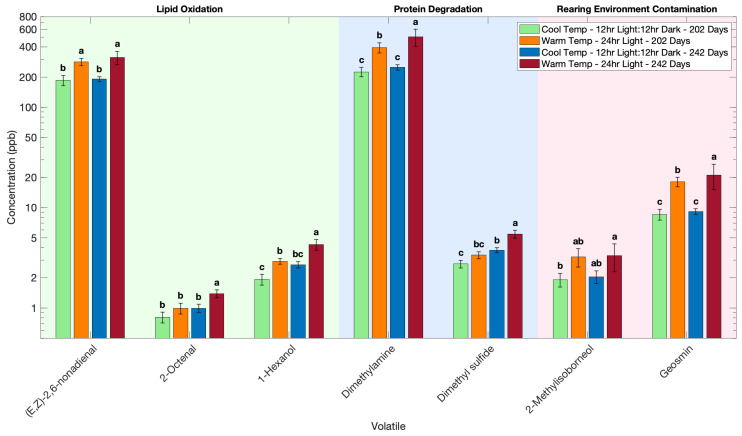
Effect of harvest time, rearing temperature, and light on the volatile profile of Atlantic salmon. Volatile concentration is plotted on a base-10 logarithmic (log_10_) scale. Different letters indicate significant difference within each volatile (*p* < 0.05).

Rearing temperature and light conditions significantly influenced the volatile profile of Atlantic salmon. All the measured volatiles are presented in Table A1, with key volatiles from each category highlighted in Figure 3. Rearing Atlantic salmon in warm temperatures with continuous light resulted in significantly higher volatile concentrations compared to cool temperatures with a 12 h light–12 h dark cycle. (Figure 3, Table A1). Under the warm temperature with continuous light conditions, 24 to 39 volatile compounds were significantly higher, depending upon their time of harvest (Table A1). The difference in volatile concentrations is likely due to the difference in growth and metabolism of Atlantic salmon under these temperature and light conditions.

Volatiles derived from lipid oxidation were significantly higher in the warm temperature with continuous light condition (left side of Figure 3). (E,Z)-2,6-Nonadienal, known for its cucumber-like aroma, related to spoilage by lipid oxidation in fish [25], was at higher concentrations in the warm temperature with continuous light condition than in the cool temperature with 12 h light–12 h dark cycle conditions. A similar pattern was observed for 2-octenal, an indicator of rancid lipid oxidation [26], and 1-hexanol, an alcohol from lipid hydroperoxide breakdown [27]. Fish raised in the warm temperature with continuous light regime, which was near the upper thermal tolerance for Atlantic salmon [28,29,30], showed accelerated lipid oxidation, leading to higher concentrations of aldehyde and alcohol volatiles. Warmer, continuous-light conditions can elevate the fish’s metabolic rate, thereby predisposing tissues to oxidative degradation. Fast-growing fish generally have higher metabolic oxygen demand and generate more reactive oxygen species (ROS) [31,32], and if antioxidant defenses are depleted, lipid peroxidation will increase. In this study, fish reared in warmer, continuous-light conditions grew more rapidly, as expected, due to the higher temperature and 24 h light, which likely contributed to a pro-oxidative muscle environment (Figure 2). Rapid growth and higher rearing temperatures with continuous light exposure diminishes fillet antioxidant levels in Atlantic salmon [33,34,35,36,37,38,39,40]. Under continuous light, Atlantic salmon retain less astaxanthin than those reared with a 12 h light–12 h dark cycle, an effect attributed to oxidative stress [33,34,35,36,37,38,39,40]. This depletion of antioxidants means that lipids in the fish reared at warm temperature with continuous light were more prone to oxidation, aligning with the increased concentration of (E,Z)-2,6-nonadienal, 2-octenal, and 1-hexanol we observed. Increased total body weight and length in the warm temperature with continuous-light regime, compared to cool temperature (Figure 2), indicate enhanced growth, which suggests an increase in oxidative volatile formation, whereas the cooler temperature with 12 h light–12 h dark cycle regime resulted in lower total body weight and length, leading to a lower baseline of lipid oxidation products.

Atlantic salmon reared in the warm temperature with continuous-light regime also showed higher levels of volatiles associated with protein and nucleotide breakdown (middle of Figure 3). Dimethylamine (DMA) was more than two-fold higher in the warm temperature with continuous-light regime, as compared to the cold temperature with 12 h light–12 h dark cycle. Dimethylamine is a common spoilage indicator that arises from the bacterial or enzymatic reduction of trimethylamine N-oxide (TMAO) in seafood [41]. Its elevation in the warm temperature with continuous-light regime suggests that these fish underwent more extensive post mortem deamination and spoilage. This could be due to the rapid onset of autolysis in stress-affected muscle at higher temperatures. Likewise, dimethyl sulfide (DMS), a volatile sulfur compound often linked to microbial degradation of sulfur-containing amino acids or osmolytes [42], was also higher in fish reared in warm temperature with continuous light. The fish reared in cooler temperature with 12 h light–dark cycle regime, by contrast, maintained very low dimethyl sulfide and dimethylamine levels, consistent with a fresher fillet with minimal spoilage. These results imply that fillets from fish reared in the warm temperature with continuous-light regime were biochemically older at harvest. This is an outcome likely related to chronic rearing stress, which will result in reduced flesh quality and shelf-life [32]. In Atlantic salmon, high rearing temperatures can accelerate quality deterioration once the fish is harvested. Stress elevates basal metabolic activity and can exhaust energy reserves, potentially altering rigor mortis and pH decline in a manner that favors spoilage microflora. While in this study harvest protocols were kept identical in order to prevent any confounding differences, the fish grown in the warm temperature with continuous-light regime still exhibited higher endogenous breakdown of proteins (such as dimethylsulfide and dimethylamine) at harvest, pointing to the presence of higher precursors leading to off-odor production. This means Atlantic salmon raised in the warm temperature with continuous-light regime might have a shorter shelf-life and develop off-odors faster than those raised at cooler temperatures.

Volatiles originating from microbial activity such as geosmin and 2-methylisoborneol, which are terpenoid metabolites often originating from environmental microbes, were also lower in cooler temperature with 12 h light–dark cycle conditions as compared to warm temperature with continuous-light conditions (right side of Figure 3). Geosmin levels in the warm-reared fish were roughly double those found in the cool-reared fish. A similar increase was seen for 2-methylisoborneol in the warm temperature with continuous-light regime. These off-flavors are known to cause “muddy” or “musty” taints in farmed fish and are typically produced by cyanobacteria or actinomycete bacteria in the water [43]. The higher concentrations in the warm temperature with continuous-light regime suggest that environmental microbial activity or uptake of these compounds was enhanced under warm, continuous light conditions. Warmer water can stimulate the growth of geosmin and 2-methylisoborneol-producing microbes, and the lack of a dark period might have allowed any algae/bacteria in the system to proliferate [43]. Additionally, geosmin and 2-methylisoborneol accumulate in fish adipose tissue by passive partitioning [43]. The warm temperature with continuous-light regime fish had a higher lipid content (68.7 mg) than the cool temperature with 12 h light–dark cycle fish (42.7 mg lipid); thus, they would have absorbed more of these compounds from the water. Prior studies report that depuration (purging in clean water) is required to remove these volatiles [43]. In this study, the fish raised in warm conditions were in an open flow-through system with a much higher rate of water exchange compared to the fish in cool conditions, where the water was recirculated prior to analysis. This finding is important because even trace levels of these terpenoids can negatively impact consumer perception due to their earthy off-odors. Contrary to expectations, fish reared under a cool temperature with a recirculating aquaculture system exhibited significantly lower levels of off-flavor volatiles such as geosmin and 2-methylisoborneol as compared to warm temperature with an open water system. The cool temperature used recirculated water, which could have concentrated off-odors; however, the lower temperature and 12 h light–dark cycle regime limited off-flavor uptake, likely by keeping microbial growth in check and possibly by producing fillets with less of a reservoir for hydrophobic taints.

Although this study did not independently vary the photoperiod, it is worth noting that constant light exposure may have accelerated the effects of high temperature. Continuous light prevents normal circadian rhythms in Atlantic salmon, which can increase stress hormone levels and disrupt metabolic regulation [44]. However, photoperiod alone has a relatively modest impact on flesh quality compared to temperature extremes [35,45]. Imsland et al. (2019) found that switching between 18 h and 24 h light had only minor effects on fillet texture and composition, whereas rearing temperature differences had significant impacts [45]. In our study, the 12 h light–dark cycle at cooler temperature likely helped the fish maintain normal diurnal rhythms, partially shielding them from oxidative damage [46]. In contrast, the continuous light regimen at warmer temperature likely imposed continuous metabolic activity [35,46]. The lack of a rest period could have intensified oxidative stress and possibly immunosuppression, making the fish more susceptible to microbial colonization and hence higher spoilage volatiles [35,46]. Most of the volatile profile divergence was due to the temperature effect, with constant light mainly acting as an additional stressor.

The observed volatile differences can be explained by the profound effects of rearing temperature (and secondarily photoperiod) on Atlantic salmon physiology. Atlantic salmon are a cold-water species; 13 °C falls within their optimal growth range, whereas 20 °C is near their upper thermal limit and imposes chronic metabolic stress [28,29,34]. The 7 °C temperature gap led to very different growth rates and metabolic profiles. At 13 °C, growth was slower, and fish experienced regular nocturnal periods, conditions that favor metabolic recovery, no oxidative stress, and higher antioxidant levels in fish [28,29,34]. In contrast, the 20 °C fish were pushed to maximize growth under continuous light. Such fast growth comes at a cost, as it elevates the production of ROS and can overwhelm antioxidant defenses [28,29,34,35]. Our findings are in line with [28,29,32,34,35], who reported that fish growing at higher temperatures and in continuous light exhibit higher oxidative stress and lower total antioxidant capacity than fish growing at cooler temperatures and under natural photoperiods. Intensive growth tends to alter fillet composition, often reducing levels of antioxidants [28,35,39]. In this study, warm temperature and a continuous light regime likely created a similar oxidative challenge, which resulted in higher oxidation-derived volatiles. Additionally, faster growth can affect lipid metabolism. Some studies note that rapid growth may either increase lipid deposition or, conversely, decrease lipid percentage if muscle growth outpaces fat storage [32]. In this study, regardless of net lipid content, the warm-reared fish clearly had a greater fraction of unstable polyunsaturated lipids oxidized, as evidenced by aldehydes like 2,6-nonadienal. Therefore, the rearing conditions that promoted the most growth also promoted the most chemical degradation of the fillet.

### 3.2. Effect of Flipping Rearing Conditions on the Volatile Profile of Atlantic Salmon

We observed that raising Atlantic salmon at higher temperatures under continuous light conditions leads to a sharp increase in volatile compounds responsible for off-odors. Warm conditions are ideal for fast growth and quickly achieved market size of Atlantic salmon, but this rearing condition can also compromise flavor quality. So, we explored whether changing rearing conditions near the end of growth, by flipping from warm to cool or cool to warm rearing conditions, could help reduce the undesirable volatiles and improve the fish’s overall flavor.

**Figure 4:**
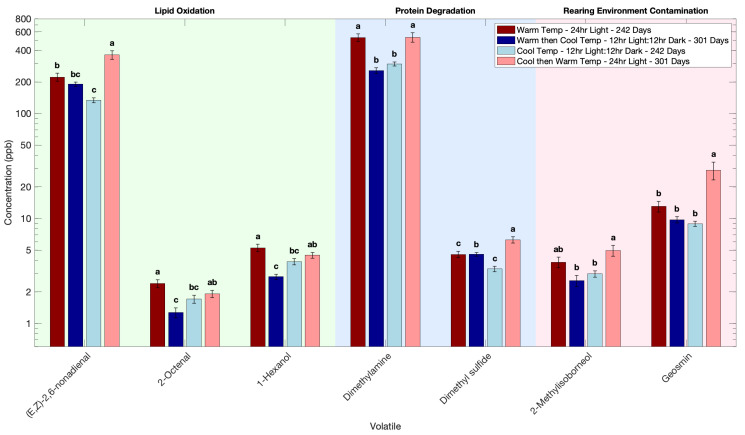
Effect of flipping rearing temperature and light conditions on the volatile profile of Atlantic salmon. Volatile concentration is plotted on a base-10 logarithmic (log_10_) scale. Different letters indicate significant difference within each volatile (*p* < 0.05).

Changing the fish grown in a warm rearing temperature with 24 h light exposure to cool temperature with a 12 h-light–12 h-dark cycle, significantly decreased 32 volatile compounds (Table A2). The lipid oxidation volatiles (E,Z)-2,6-nonadienal, 1-hexanol, and 2-octenal were reduced after switching to the cooler rearing conditions with a 12 h light– 12h-dark cycle (Figure 4). Volatiles generated from protein degradation, such as dimethylamine, were also reduced (Figure 4). The earthy and musty note-producing volatiles geosmin and 2-methylisoborneol were also lower, suggesting that moving fish grown in warm conditions into cooler 12:12 light–dark conditions favors a decrease in off-odor producing volatiles (Figure 4).

Changing the fish grown in cooler conditions with a 12 h-light–12 h-dark cycle to warm rearing temperature and 24 h light exposure, the effects were reversed (Figure 4, Table A2). A total of 36 volatiles increased significantly, including many of the same off-odor volatile compounds that had decreased when moving from warm rearing temperature with 24 h light exposure to cool temperature with a 12 h-light–12 h-dark cycle (Figure 4, Table A2). This confirmed that warmer temperatures and extended light exposure increased the metabolic activity that drives volatile compounds associated with spoilage and off-odors, leading to higher volatile compound production, whereas cooler conditions with a 12 h-light–12 h-dark cycle lowered metabolic activity, leading to lower volatile compound formation.

These results suggest a promising strategy to grow fish most efficiently under warm conditions until they reach market size, then flip to cooler conditions shortly before harvest to reduce off-odor producing volatiles. This could lead to a noticeably cleaner and more appealing flavor in the final product. This kind of temperature flipping could be an easy and cost-effective way to improve flavor quality in farmed Atlantic salmon without major changes to farming infrastructure.

### 3.3. Effect of Harvest Time on the Volatile Profile of Atlantic Salmon

The harvest age of Atlantic salmon had a significant impact on the volatile profile. Older fish, harvested at 242 days, consistently showed higher concentrations of volatile compounds than those harvested earlier, at 202 days (Figure 2 and Table A1). There was an interaction with rearing temperature, as fish grown at cooler temperature showed higher levels of only 5 volatiles, whereas fish grown at warm temperature showed higher levels of 22 volatiles at the later harvest (Table A1). There was no change in the concentrations of other volatiles with growth time.

As the fish continue to grow, they mature physiologically, which appears to create higher concentrations of volatiles. By 242 days, the Atlantic salmon had greater total body weight, longer length, larger gonads, and a higher gonadosomatic index (GSI) (Table 2). As the fish matures, there is a higher accumulation of biochemical components including lipids and proteins in fish tissue [47,48,49,50], which act as substrates for post mortem degradation processes that generate volatiles. In particular, the higher fat reserves in later harvested Atlantic salmon [47,48,49,50] fuel more extensive lipid oxidation after harvest, producing larger amounts of volatiles such as aldehydes and alcohols [32,51,52].

The volatiles 2-octenal and (E,Z)-2,6-nonadienal are formed from polyunsaturated fatty acid oxidation, and they were higher in older fish than in younger fish, consistent with the theory that fish that contain more fat tend to undergo a greater degree of oxidation and flavor compound formation during storage (Figure 2). Likewise, 1-hexanol from fatty acid oxidation was higher in older fish, reflecting the more advanced oxidative breakdown of lipids when a larger lipid pool is present. These findings align with general observations that fish with higher fat content develop more oxidation byproducts and hence volatiles during post-harvest storage, as the fat-rich tissues generate rancid off-flavors more readily upon exposure to oxygen [32,47,48,49,50,51,53].

In addition to lipid-driven changes, the greater protein content in older Atlantic salmon contributes to higher levels of amine and sulfur volatiles after harvest [54,55]. As Atlantic salmon approach maturation, hormonal and metabolic changes can alter muscle composition, which in turn may affect post-harvest microbial growth and enzyme activity [53,54,55,56]. Our results support this theory as the older, more mature Atlantic salmon had a volatile profile indicative of more advanced protein degradation, whereas younger fish maintained lower levels of these breakdown products (Figure 2). After harvest, trimethylamine N-oxide in the muscle is broken down by endogenous enzymes and microbes, yielding the volatiles dimethylamine and dimethyl sulfide [41,57]. The older fish showed notably higher concentrations of dimethylamine and dimethylsulfide (Figure 2), suggesting an accelerated generation of these volatiles from protein degradation as compared to the younger fish. This agrees with prior studies identifying maturity as a factor that can decrease fish flavor and freshness over time after harvest [32,47,48,49,50,51,52,53].

Another important reason the older fish accumulated more volatiles is their longer exposure to environmental contaminants prior to harvest. Off-flavor terpenoids originating from the aquaculture environment, geosmin and 2-methylisoborneol [58,59,60], were higher in older fish (Figure 2). Because these compounds are absorbed from the water and stored in fish lipid tissues, their levels depend on both water concentration and exposure duration [60,61]. The older Atlantic salmon spent an extra 38 days in the system, giving them additional time to uptake and bioaccumulate geosmin and 2-methylisoborneol from feed or water. Moreover, older fish have larger lipid stores to sequester these hydrophobic compounds. This explains the consistently greater geosmin and 2-methylisoborneol concentrations at 242 days, even though the fish were kept in the same environment as those harvested at 204 days. The time of exposure, size, and age of the fish are major factors determining off-flavor producing compounds in cultured fish [60,61]. In this study, the older and bigger Atlantic salmon had higher amounts of off-flavor producing volatiles, in agreement with those principles.

### 3.4. Effect of Sex on the Volatile Profile of Atlantic Salmon

**Figure 5:**
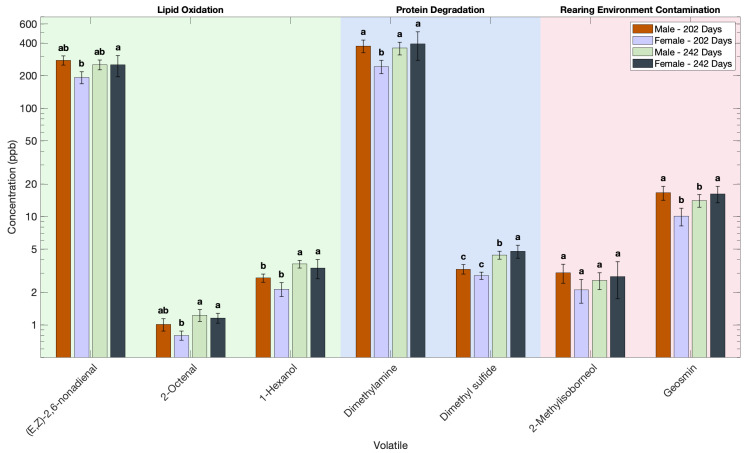
Effect of sex on the volatile profile of Atlantic salmon. Volatile concentration is plotted on a base-10 logarithmic (log_10_) scale. Different letters indicate significant difference in each volatile (*p* < 0.05).

The volatile profile of Atlantic salmon is significantly affected by the sex of the fish (Figure 5, Table A3), reflecting physiological and biochemical differences established prior to harvest. At the earlier harvest, male Atlantic salmon exhibited higher concentrations of 19 volatile compounds compared to females (Figure 5, Table A3). This difference can be attributed to the males’ biological focus on somatic growth and general metabolism during early juvenile development. Supporting this, gonadosomatic index (GSI) values and gonadal weight indicated that males had small, immature gonads and were still in a pre-pubertal stage at this time, likely resulting in the accumulation of various biochemical precursors contributing to post mortem volatile formation (Table 2). In contrast, females at this stage were ahead in sexual development and had already initiated early gonad development, suggesting a gradual metabolic shift toward reproductive investment.

By the later harvest, a reversal in the volatile profile was observed; female Atlantic salmon produced significantly higher concentrations of 13 volatile compounds compared to males (Figure 5, Table A3). At this stage, both sexes exhibited increased gonad size. However, females had substantially larger gonadal weights and higher GSI values (Table 2), indicating more advanced reproductive development. This physiological transition in females is closely associated with sexual maturation, which involves substantial lipid mobilization and protein turnover processes known to contribute to the increased generation of volatile compounds following harvest.

Lipid-derived volatiles are the major contributors to sex-specific differences in the volatile profile of Atlantic salmon. Lipid oxidation, especially of polyunsaturated fatty acids, leads to hydroperoxide formation and subsequent breakdown into volatile aldehydes, ketones, and alcohols [62]. During early juvenile stages, male Atlantic salmon invest heavily in somatic growth, resulting in the accumulation of energy-dense lipids such as triacylglycerols and membrane phospholipids like phosphatidylcholines in muscle tissues [63]. These lipids act as substrates for post mortem oxidation, producing higher levels of aldehydes, ketones, and alcohols at the earlier harvest. As females mature their tissues become increasingly enriched in polyunsaturated fatty acids such as eicosapentaenoic acid and docosahexaenoic acid compounds, which are highly susceptible to oxidation [63]. This metabolic shift provides a greater pool of oxidation-prone substrates, explaining the increased abundance of lipid-derived volatiles detected in female Atlantic salmon at the later harvest. Comparative studies on other aquatic species provide further support for these observations. Zhao et al. (2024) reported that female giant salamanders, which have higher lipid levels, produced more lipid-derived volatiles than males [17]. Similarly, Jin et al. (2024) observed that female salamander liver tissues enriched in unsaturated lipids yielded significantly higher levels of alcohols and ketones [64]. These findings reinforce that sex-associated biochemical shifts, particularly in lipids, influence the volatile profiles of fish.

Protein-derived volatiles also significantly contributed to the volatile profile of Atlantic salmon through degradation after harvest. Endogenous proteases such as cathepsins and calpains degrade structural proteins, liberating peptides and amino acids, which undergo further breakdown into volatile amines, thiols, and sulfides [62,65]. At the earlier harvest, male Atlantic salmon also showed higher levels of volatiles, which aligns with their higher structural protein content during early growth. A study on largemouth bass demonstrated that males possess greater concentrations of certain amino acids and collagen, contributing to a stronger muscle matrix and potentially greater post mortem protein breakdown, than females [66]. As females mature, muscle protein is increasingly catabolized to supply amino acids for ovary development. This is supported by findings in Atlantic salmon showing muscle protein trnover during reproductive maturation [67]. The resulting free amino acids, especially sulfur- and nitrogen-containing ones such as methionine and lysine, are precursors to amines, ammonia, and sulfur-containing volatiles, contributing to the complex post-harvest off-odors in maturing female Atlantic salmon.

Geosmin is a lipophilic terpenoid that accumulates in fatty tissues during the life of the fish and is released post mortem as tissue structures break down [43]. Male Atlantic salmon at 202 days and female Atlantic salmon at 242 days had a significantly higher geosmin concentration than female Atlantic salmon at 202 days and male Atlantic salmon at 242 days. This suggests that males at 202 days and females at 242 days may have higher fat content, which lead to greater geosmin accumulation and release after post-harvest muscle breakdown. The same trend was shown by 2-methylisoborneol, but the differences were not significant.

### 3.5. Effect of Skin Presence on the Volatile Profile of Atlantic Salmon

**Figure 6:**
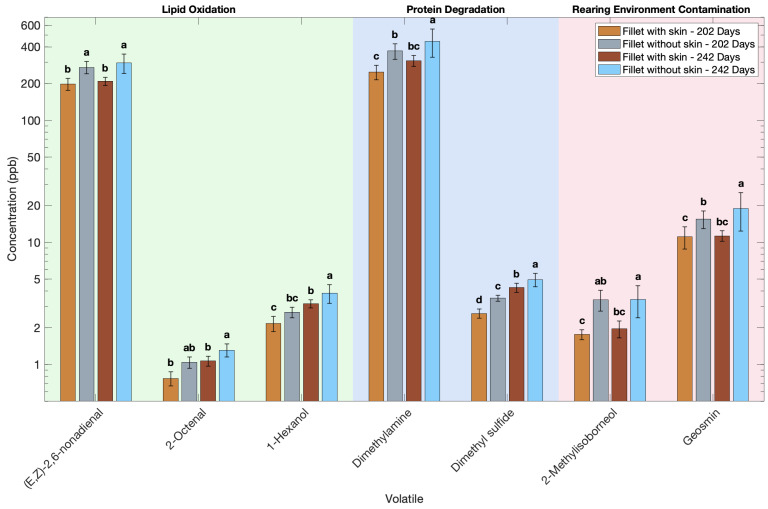
Effect of presence of skin on the volatile profile of Atlantic salmon fillets. Volatile concentration is plotted on a base-10 logarithmic (log_10_) scale. Different letters indicate significant difference in each volatile (*p* < 0.05).

To determine the effect of skin on volatile production in the muscle, two types of samples were analyzed—fillet without skin (muscle only) and fillet with skin (muscle plus skin). The samples were frozen at −80 °C for 30 days before analysis. The presence of skin significantly influenced the volatile profile in Atlantic salmon fillets. At both harvest times (202 and 242 days), fillets with skin consistently exhibited higher concentrations of almost 40 volatile compounds compared to fillets without skin (Figure 6, Table A4). The same trend was observed at both harvests, strengthening the conclusion that the increase in volatile concentration is caused by the presence of the skin. These findings align with previous studies in other fish species, where skin portions were found to contain significantly higher total volatile levels than corresponding muscle samples [22,68]. The skin-only portion showed higher levels than muscle for most of the volatiles. The presence of skin appears to increase both the formation and retention of volatile organic compounds in the frozen stored fillets.

Volatiles derived from lipid oxidation increased significantly in fillets with skin as compared to fillets without skin (Figure 6). Atlantic salmon skin and its adjoining tissues, including subcutaneous fat and the underlying dark muscle, are rich in lipids [69], particularly unsaturated fatty acids that serve as precursors for lipid oxidation volatiles. In Atlantic salmon, skin can contain nearly twice the lipid content as muscle, creating a substantial reservoir of oxidizable substrates [70,71]. The presence of heme pigments and iron in the dark muscle further catalyzes lipid peroxidation, facilitating the formation of aldehydes [68,72].

Volatiles derived from protein degradation also increased significantly in fillets with skin as compared to fillets without skin (Figure 6). Fillets with skin demonstrated significantly higher levels of dimethylamine and dimethylsulfide. The skin surface and subdermal region are biochemically active zones, harboring dense microbial populations that can generate volatile metabolites [73,74,75,76,77]. These microbes can degrade compounds such as trimethylamine N-oxide, generating volatile amines like trimethylamine and dimethylamine—major contributors to fishy odors [78,79,80]. The proteins are bound to tissues in fish. Upon freezing, damage to the tissue results in the release of proteins from the damaged tissue, which leads to more precursors for protein degradation during thawing, as the activity of microbes slows down while frozen but increases as the temperature increases, and this leads to higher amounts of protein-derived volatiles. The removal of skin, therefore, not only reduces the microbial load but also slows down the generation of spoilage-associated volatiles.

Geosmin and 2-methylisoborneol both were significantly higher in fillets with skin as compared to fillets without skin (Figure 6). These terpenoid compounds preferentially accumulate in fatty tissues such as the skin and the adjacent dark muscle [43], and because of this, both were higher in muscle with skin, suggesting that skin removal also eliminates a considerable portion of pre-existing off-odor agents.

All Atlantic salmon fillets in this study were stored frozen prior to analysis, a process known to alter cellular integrity. Freezing leads to ice crystal formation that disrupts muscle cell membranes and results in tissue damage [81,82,83]. Damaged tissue results in the leakage of lipids, proteins, and enzymes into the surrounding matrix, which further leads to lipid oxidation, protein breakdown, and the release of other off-odor producing volatiles. In fillet with skin samples, the effect of freezing is higher due to the higher lipid content from the skin along with the adjacent dark muscle. Thus, structural damage from freezing leads to an increase in the generation of volatile compounds from the biochemical composition of both muscle and skin.

## 4. Conclusions

The rearing, physiological, and processing conditions significantly influence the volatile profile of Atlantic salmon, ultimately affecting fillet quality, freshness, and flavor perception. Rearing conditions such as temperature and light exposure had the greatest effect on volatile levels of any of the parameters measured in this study. Fish reared at a warm temperature under continuous light developed significantly higher levels of volatiles producing off-odors. These changes were attributed to elevated metabolic activity, increased oxidative stress, and microbial proliferation under thermally and photoperiodically stressful conditions. However, Atlantic salmon reared at a cooler temperature with a 12 h light–dark cycle exhibited a fresher volatile profile, highlighting the benefits of cooler, circadian-aligned environments in maintaining fillet freshness. Atlantic salmon reared at cooler temperatures and under a natural 12 h light–dark cycle produce fewer off-odor volatiles, making these conditions ideal for maximizing Atlantic salmon quality. A potential strategy for improving flavor in farmed Atlantic salmon is to raise the fish under warm conditions with continuous light for faster growth, then switch to cooler temperatures with a 12 h–light dark cycle shortly before harvest to reduce off-flavor volatiles. This temperature-flipping approach could enhance flavor quality in a simple and cost-effective way without major changes to current farming practices.

Harvest time also had an impact, with Atlantic salmon harvested later exhibiting consistently higher concentrations of volatiles than those harvested earlier. This increase is linked to physiological maturity and higher biochemical composition, particularly greater lipid and protein content in older fish, which enhances the substrate availability for post mortem oxidation and microbial degradation processes. Sex also showed dynamic and maturation-dependent effects on volatile profile. At the early harvest, male Atlantic salmon showed higher volatile concentrations due to early biochemical deposition associated with the onset of puberty. By the later harvest, females surpassed males in volatile concentration, likely due to rapid biochemical accumulation in preparation for reproduction. The increase in formation of spoilage-related volatiles suggest the importance of optimizing harvest time. Sex-related differences in growth, metabolism, and reproductive investment influence off-odor-related volatiles over time. Tailoring harvesting strategies based on fish maturity and sex could also help deliver more consistent and appealing volatile profiles. The combination of these mechanisms contributes to a complex matrix of volatiles that varies by sex, harvest time, and physiological status. Our results suggest that the volatile profile depends on both harvest time and sex, based on growth and maturation stage shaping the sensory and chemical characteristics of Atlantic salmon. Recognizing these trajectories is essential for optimizing harvest timing, improving product quality, and tailoring aquaculture strategies to align with desired sensory outcomes. The post mortem volatile profile serves as a reliable biomarker of a fish’s physiological history and can inform quality grading and processing decisions.

Finally, the presence of skin on the fillet significantly increased the levels of off-odor-related volatiles. This was due to the skin’s high biochemical content along with muscle. This biochemical content acted as a source of lipid oxidation, protein degradation precursors, and accumulated lipophilic off-flavor compounds like geosmin and 2-methylisoborneol. These results suggest that post-harvest handling practices, such as filleting with or without skin, can have a measurable impact on the sensory properties and shelf-life of Atlantic salmon. Removing the skin prior to freezing is advisable for products destined for long-distance transport or prolonged storage to minimize oxidation and microbial spoilage.

This research provides critical insight into the volatiles responsible for off-odor development in Atlantic salmon and offers actionable solutions to control their formation. It establishes that rearing temperature, light exposure, harvest time, sex, and the presence of skin all influence the biochemical pathways that generate spoilage-related volatiles. These findings not only enhance consumer acceptability but can also contribute to waste reduction by minimizing early spoilage and product rejection. Integrating this knowledge into aquaculture practices and processing workflows can help the industry deliver higher quality seafood with extended shelf-life, improved traceability of quality issues, and greater market competitiveness. Future research should expand on exploring the impact of feed composition, on volatile profile of fish. Longitudinal studies that track volatile accumulation across multiple life stages would offer deeper insights into off-flavor dynamics over time. Investigating interventions such as antioxidant supplementation could further mitigate undesirable aroma compounds.

## Figures and Tables

**Table 1:** Properties of volatiles tested in selected-ion flow-tube mass spectrometer (SIFT-MS) headspace analysis.

Volatile	Reagent	k (10^−9^ cm^3^/s)	Mass (*m*/*z*)	Product
(E,Z)-2,6-nonadienal	NO^+^	2.5	137	C_9_H_13_O^+^
(E)-2-pentenal	NO^+^	4	83	C_5_H_7_O^+^
1-hexanol	NO^+^	2.4	101	C_6_H_13_O^+^
1-octanol	NO^+^	2.3	129	C_8_H_17_O^+^
1-octen-3-ol	H_3_O^+^	2.5	111	C_8_H_15_^+^
1-octen-3-one	NO^+^	2.5	156	C_8_H_14_.NO^+^
1-pentanol	H_3_O^+^	2.8	71	C_5_H_11_^+^
1-penten-3-ol	H_3_O^+^	2.6	69	C_5_H_9_^+^
2-butenal	NO^+^	4.1	69	C_4_H_5_O^+^
2-decanone	NO^+^	2.5	186	C_10_H_20_O.NO^+^
2-decenal	NO^+^	2.1	153	C_10_H_17_O^+^
2-heptanone	NO^+^	3.4	144	C_7_H_14_O.NO^+^
2-heptenal	H_3_O^+^	4.7	113	C_7_H_13_O^+^
			131	C_7_H_13_O^+^.H_2_O
2-hexenal	H_3_O^+^	4.6	99	C_6_H_11_O^+^
			117	C_6_H_11_O^+^.H_2_O
2-methyl naphthalene	H_3_O^+^	2.5	143	C_11_H_11_^+^
2-nonanone	NO^+^	2.7	172	C_9_H_18_O.NO^+^
2-nonenal	H_3_O^+^	4.8	141	C_9_H_17_O^+^
			159	C_9_H_17_O^+^.H_2_O
2-octenal	NO^+^	4.1	125	C_8_H_13_O^+^
2-octene	NO^+^	2.1	112	C_8_H_16_^+^
2-pentanone	NO^+^	3.1	116	NO^+^.C_5_H_10_O
2-penten-1-ol	H_3_O^+^	3	69	C_5_H_9_^+^
2-pentene	H_3_O^+^	1.9	71	C_5_H_11_^+^
2-undecanone	NO^+^	3.4	200	C_11_H_22_O.NO^+^
2,4-decadienal	NO^+^	4.2	151	C_10_H_15_O^+^
2,4-heptadienal	NO^+^	2.1	57	C_3_H_5_O^+^
3-hexen-1-ol	H_3_O^+^	3.2	83	C_6_H_11_^+^
3-hexenal	H_3_O^+^	4.2	81	C_6_H_9_^+^
decanal	NO^+^	3.3	155	C_10_H_19_O^+^
heptanal	NO^+^	3.3	113	C_7_H_13_O^+^
heptane	H_3_O^+^	2.6	119	H_3_O^+^.C_7_H_16_
hexanal	NO^+^	2.5	99	C_6_H_11_O^+^
hexanoic acid	NO^+^	2.5	146	C_6_H_12_O_2_.NO^+^
nonanal	NO^+^	2.7	141	C_9_H_17_O^+^
oct-2-en-1-ol	H_3_O^+^	2.8	111	C_8_H_14_.H^+^
octanal	NO^+^	3	127	C_8_H_15_O^+^
octane	H_3_O^+^	9	113	C_8_H_17_^+^
pentanal	NO^+^	3	85	C_5_H_9_O^+^
pentane	O_2_^+^	1.6	43	C_3_H_7_^+^
propanal	NO^+^	2.5	57	C_3_H_5_O^+^
trans-2-undecenal	H_3_O^+^	3	169	C_11_H_20_O.H^+^
2-isopropyl-3-methoxypyrazine	H_3_O^+^	3	153	C_8_H_12_N_2_O.H^+^
2-methylisoborneol	H_3_O^+^	2.9	151	C_11_H_19_^+^
2,3-butanediol	NO^+^	2.3	89	C_4_H_9_O_2_^+^
			107	C_4_H_9_O_2_^+^.H_2_O
alpha-terpinene	NO^+^	2	136	C_10_H_16_^+^
beta-caryophyllene	NO^+^	2.7	204	C_15_H_24_^+^
ethyl acetate	NO^+^	2.7	118	NO^+^.CH_3_COOC_2_H_5_
geosmin	NO^+^	2.5	112	C_8_H_16_^+^
isobutyl alcohol	NO^+^	2.4	73	C_4_H_9_O^+^
3-methyl-1-butanol	H_3_O^+^	2.8	71	C_5_H_11_^+^
acetic acid	NO^+^	9	90	NO^+^.CH_3_COOH
acetoin	NO^+^	3	118	C_4_H_8_O_2_.NO^+^
acetone	NO^+^	1.2	88	NO^+^.C_3_H_6_O
carbon disulfide	O_2_^+^	4	76	CS_2_^+^
dimethyl disulfide	NO^+^	2.4	94	(CH_3_)_2_S_2_^+^
dimethyl sulfide	NO^+^	2.2	62	(CH_3_)_2_S^+^
formaldehyde	H_3_O^+^	3.4	31	CH_3_O^+^
			49	H_2_CO.H^+^.H_2_O
hydrogen sulfide	H_3_O^+^	1.6	35	H_3_S^+^
indole	H_3_O^+^	3.3	118	C_8_H_8_N^+^
methyl mercaptan	H_3_O^+^	1.8	49	CH_4_S.H^+^
trimethylamine	NO^+^	1.6	59	(CH_3_)_3_N^+^
ammonia	H_3_O^+^	2.6	18	NH_4_^+^
			36	NH_4_^+^.H_2_O
dimethylamine	H_3_O^+^	2.1	46	(CH_3_)_2_NH.H^+^
2-acetyl furan	NO^+^	2	110	C_6_H_6_O_2_^+^
2-ethyl-2,5, dimethylpyrazine	O_2_^+^	2.5	136	C_8_H_12_N_2_^+^
2-ethylfuran	NO^+^	2.9	96	C_6_H_8_O^+^
trimethylpyrazine	NO^+^	2.5	122	C_7_H_10_N_2_^+^

**Table 2:** Effect of harvest time, weight, length, and sex on gonads and gonadosomatic index (GSI) of Atlantic salmon. Different letters in each factor indicate significant difference in each factor (*p* < 0.05).

Harvest Time	Total Body Weight (g)	Length (cm)	GSI (%)	
			Male	Female
202 Days	161 ^b^ ± 16.2	26.4 ^b^ ± 0.54	0.00 ± 0	0.25 ± 0.02
242 Days	256 ^a^ ± 32.9	29.1 ^a^ ± 0.84	0.18 ± 0.08	0.27 ± 0.04

## Data Availability

The data presented in the study is available in Appendix A. Further inquiries can be directed to the corresponding author.

## Appendix A

**Table A1:** Effect of rearing temperature and light on the volatile profile of Atlantic salmon. Different letters indicate significant difference within each volatile (*p* < 0.05).

Volatile	Cool Temp 12 h L:12 h D (204 Days)	Cool Temp 12 h L:12 h D (242 Days)	Warm Temp 24 h L (204 Days)	Warm Temp 24 h L (242 Days)
(E, Z)-2,6-nonadienal	186 ^b^	191 ^b^	284 ^a^	314 ^a^
(E)-2-pentenal	93.2 ^b^	95.1 ^b^	129 ^a^	141 ^a^
1-hexanol	1.93 ^c^	2.70 ^bc^	2.93 ^b^	4.29 ^a^
1-octanol	1.79 ^c^	2.16 ^bc^	2.72 ^ab^	2.57 ^a^
1-octen-3-ol	343 ^c^	339 ^c^	654 ^b^	833 ^a^
1-octen-3-one	0.79 ^a^	0.53 ^a^	0.78 ^a^	0.86 ^a^
1-pentanol	6.48 ^b^	8.70 ^ab^	8.89 ^ab^	10.8 ^a^
1-penten-3-ol	8.75 ^b^	10.2 ^ab^	10.73 ^ab^	12.0 ^a^
2-butenal	1.32 ^c^	1.78 ^ab^	1.65 ^bc^	2.02 ^a^
2-decanone	1.24 ^a^	0.98 ^ab^	1.04 ^b^	0.97 ^ab^
2-decenal	1.78 ^b^	1.97 ^b^	3.02 ^a^	2.69 ^a^
2-heptanone	5.27 ^ab^	4.63 ^b^	5.49 ^ab^	5.96 ^a^
2-heptenal	8.94 ^c^	8.89 ^c^	12.0 ^b^	13.7 ^a^
2-hexenal	8.33 ^bc^	7.45 ^c^	11.9 ^ab^	13.9 ^a^
2-methyl naphthalene	7.40 ^b^	6.76 ^b^	11.2 ^a^	10.4 ^a^
2-nonanone	0.98 ^a^	0.87 ^a^	1.24 ^a^	1.03 ^a^
2-nonenal	38.4 ^c^	36.2 ^c^	71.2 ^b^	91.0 ^a^
2-octenal	0.81 ^b^	1.00 ^b^	0.99 ^b^	1.39 ^a^
2-octene	5.35 ^b^	5.59 ^b^	7.63 ^b^	9.60 ^a^
2-pentanone	1.00 ^b^	1.03 ^b^	1.39 ^a^	1.26 ^ab^
2-penten-1-ol	6.45 ^b^	7.53 ^ab^	7.91 ^ab^	8.82 ^a^
2-pentene	9.59 ^b^	12.9 ^ab^	13.2 ^ab^	16.0 ^a^
2-undecanone	0.79 ^c^	0.47 ^b^	0.76 ^b^	0.99 ^a^
2,4-decadienal	0.60 ^a^	0.49 ^a^	0.58 ^a^	0.70 ^a^
2,4-heptadienal	25.1 ^ab^	20.2 ^b^	29.1 ^a^	21.7 ^b^
3-hexen-1-ol	1974 ^b^	1932 ^b^	2627 ^a^	2786 ^a^
3-hexenal	3.53 ^a^	3.33 ^a^	3.68 ^a^	4.28 ^a^
decanal	1.80 ^a^	1.63 ^a^	1.61 ^a^	1.74 ^a^
heptanal	4.36 ^ab^	3.75 ^b^	5.03 ^a^	3.99 ^ab^
heptane	198 ^b^	244 ^b^	267 ^b^	390 ^a^
hexanal	5.84 ^a^	4.78 ^b^	6.43 ^a^	5.82 ^ab^
hexanoic acid	0.88 ^b^	0.90 ^b^	1.14 ^ab^	1.17 ^a^
nonanal	11.3 ^c^	11.0 ^c^	18.8 ^b^	19.5 ^a^
oct-2-en-1-ol	296 ^c^	293 ^c^	564 ^b^	718 ^a^
octanal	2.22 ^a^	2.34 ^a^	2.41 ^a^	2.51 ^a^
octane	37.5 ^c^	36.1 ^c^	47.4 ^b^	52.7 ^a^
pentanal	4.61 ^b^	6.65 ^a^	4.81 ^b^	6.11 ^a^
pentane	2324 ^b^	3081 ^ab^	3087 ^ab^	3702 ^a^
propanal	16.9 ^ab^	13.6 ^b^	19.6 ^a^	14.6 ^b^
trans-2-undecenal	5.79 ^c^	5.72 ^c^	8.58 ^b^	10.4 ^a^
2-isopropyl-3-methoxypyrazine	5.47 ^c^	4.91 ^c^	8.96 ^b^	9.19 ^a^
2-methylisoborneol	1.92 ^b^	2.05 ^ab^	3.23 ^ab^	3.32 ^a^
2,3-butanediol	40.4 ^b^	61.2 ^a^	53.3 ^ab^	66.1 ^a^
alpha-terpinene	1.62 ^a^	1.65 ^a^	1.51 ^a^	1.78 ^a^
beta-caryophyllene	0.54 ^a^	0.86 ^a^	0.97 ^a^	0.75 ^a^
ethyl acetate	4.89 ^ab^	4.42 ^b^	4.66 ^ab^	5.05 ^a^
geosmin	8.58 ^c^	9.14 ^c^	18.1 ^b^	21.2 ^a^
isobutyl alcohol	8294 ^b^	8093 ^b^	11,404 ^a^	11,873 ^a^
3-methyl-1-butanol	16.3 ^b^	18.5 ^b^	22.5 ^ab^	23.0 ^a^
acetic acid	7.03 ^b^	9.67 ^ab^	9.30 ^ab^	11.2 ^a^
acetoin	4.40 ^ab^	3.98 ^b^	4.19 ^ab^	4.55 ^a^
acetone	9.37 ^c^	12.5 ^b^	13.6 ^b^	16.8 ^a^
carbon disulfide	1004 ^c^	932 ^c^	1562 ^b^	1727 ^a^
dimethyl disulfide	4820 ^b^	4638 ^b^	7488 ^a^	8032 ^a^
dimethyl sulfide	2.75 ^c^	3.76 ^b^	3.37 ^bc^	5.45 ^a^
formaldehyde	1197 ^b^	1148 ^b^	1429 ^a^	1423 ^a^
hydrogen sulfide	0.80 ^a^	0.72 ^a^	0.55 ^a^	0.76 ^a^
indole	0.71 ^a^	0.81 ^a^	0.96 ^a^	1.08 ^a^
methyl mercaptan	9265 ^b^	8915 ^b^	12,292 ^a^	12,523 ^a^
trimethylamine	21.4 ^a^	21.7 ^a^	25.7 ^a^	26.8 ^a^
ammonia	483 ^b^	560 ^b^	566 ^b^	823 ^a^
dimethylamine	225 ^c^	250 ^c^	394 ^b^	503 ^a^
2-acetyl furan	1.32 ^a^	1.24 ^a^	1.45 ^a^	1.74 ^a^
2-ethyl-2,5, -dimethylpyrazine	1.87 ^b^	1.86 ^b^	3.11 ^ab^	3.26 ^a^
2-ethylfuran	17.2 ^b^	17.1 ^b^	28.1 ^a^	30.9 ^a^
trimethylpyrazine	13.9 ^c^	12.6 ^c^	23.7 ^b^	27.9 ^a^

**Table A2:** Effect of flipping rearing temperature and light conditions on the volatile profile of Atlantic salmon. Different letters indicate significant difference within each volatile (*p* < 0.05).

Volatile	Warm Temp 24 h L (242 Days)	Warm then Cool Temp (301 Days)	Cool Temp 12 h L:12 h D (242 Days)	Cool then Warm Temp (301 Days)
(E,Z)-2,6-nonadienal	223 ^b^	362 ^a^	134 ^c^	190 ^bc^
(E)-2-pentenal	126 ^ab^	147 ^a^	78.2 ^c^	89.3 ^bc^
1-hexanol	5.26 ^a^	4.47 ^ab^	3.89 ^bc^	2.79 ^c^
1-octanol	5.17 ^a^	5.34 ^a^	5.00 ^ab^	3.90 ^b^
1-octen-3-ol	439 ^b^	1018 ^a^	211 ^b^	339 ^b^
1-octen-3-one	1.62 ^a^	1.47 ^a^	1.46 ^a^	1.35 ^a^
1-pentanol	17.3 ^a^	17.9 ^a^	13.5 ^b^	10.9 ^b^
1-penten-3-ol	27.5 ^a^	23.0 ^a^	23.8 ^a^	15.2 ^b^
2-acetyl furan	2.99 ^a^	2.06 ^b^	2.02 ^bc^	1.48 ^c^
2-butenal	3.64 ^a^	2.07 ^bc^	2.99 ^ab^	1.59 ^c^
2-decanone	2.78 ^a^	1.76 ^a^	2.09 ^a^	1.60 ^a^
2-decenal	4.79 ^a^	3.42 ^ab^	4.04 ^a^	2.20 ^b^
2-ethyl-2,5,-dimethylpyrazine	3.22 ^b^	5.24 ^a^	2.23 ^b^	2.41 ^b^
2-ethylfuran	21.8 ^b^	37.5 ^a^	13.5 ^b^	18.0 ^b^
2-heptanone	4.61 ^b^	6.81 ^a^	4.12 ^b^	5.24 ^b^
2-heptenal	14.5 ^b^	19.2 ^a^	11.1 ^b^	9.55 ^b^
2-hexenal	15.0 ^a^	17.5 ^a^	11.1 ^b^	9.25 ^b^
2-isopropyl-3-methoxypyrazine	14.7 ^a^	9.66 ^ab^	10.6 ^ab^	5.38 ^b^
2-methyl naphthalene	15.7 ^b^	38.3 ^a^	9.72 ^b^	22.2 ^b^
2-methylisoborneol	3.84 ^ab^	4.95 ^a^	2.97 ^b^	2.56 ^b^
2-nonanone	2.53 ^a^	1.78 ^a^	2.10 ^a^	1.63 ^a^
2-nonenal	42.8 ^b^	119 ^a^	23.3 ^b^	39.8 ^b^
2-octenal	2.40 ^a^	1.92 ^ab^	1.71 ^bc^	1.27 ^c^
2-octene	8.53 ^b^	12.5 ^a^	5.85 ^b^	5.54 ^b^
2-pentanone	2.01 ^a^	1.92 ^a^	1.77 ^b^	1.17 ^c^
2-penten-1-ol	20.3 ^a^	17.0 ^a^	17.6 ^a^	11.2 ^b^
2-pentene	25.6 ^a^	26.5 ^a^	19.9 ^b^	16.2 ^b^
2-undecanone	1.76 ^a^	1.63 ^a^	1.72 ^a^	1.40 ^a^
2,3-butanediol	102 ^a^	89.3 ^ab^	93.8 ^bc^	64.9 ^c^
2,4-decadienal	1.22 ^a^	0.96 ^ab^	1.01 ^ab^	0.76 ^b^
2,4-heptadienal	63.8 ^a^	20.4 ^b^	38.6 ^ab^	19.5 ^b^
3-hexen-1-ol	2651 ^a^	2952 ^a^	1782 ^b^	1799 ^b^
3-hexenal	6.80 ^a^	6.08 ^a^	5.94 ^a^	4.61 ^a^
3-methyl-1-butanol	32.1 ^a^	29.3 ^ab^	19.5 ^b^	18.7 ^b^
acetic acid	21.0 ^a^	13.9 ^b^	17.0 ^b^	10.0 ^c^
acetoin	5.64 ^a^	5.88 ^a^	4.54 ^b^	4.42 ^b^
acetone	20.8 ^a^	17.9 ^ab^	16.8 ^b^	11.9 ^c^
alpha-terpinene	2.96 ^a^	2.55 ^ab^	2.14 ^bc^	1.56 ^c^
ammonia	547 ^b^	861 ^a^	469 ^b^	564 ^b^
beta-caryophyllene	2.44 ^a^	1.43 ^ab^	2.39 ^a^	1.28 ^b^
carbon disulfide	1149 ^b^	2365 ^a^	718 ^b^	1120 ^b^
decanal	2.91 ^a^	2.90 ^ab^	2.60 ^ab^	2.00 ^b^
dimethyl disulfide	5607 ^b^	10,081 ^a^	3415 ^b^	4948 ^b^
dimethyl sulfide	4.55 ^bc^	6.27 ^a^	3.31 ^c^	4.56 ^b^
dimethylamine	531 ^a^	534 ^a^	297 ^b^	257 ^b^
ethyl acetate	6.27 ^a^	6.53 ^a^	5.05 ^b^	4.92 ^b^
formaldehyde	1507 ^b^	1659 ^a^	1291 ^c^	1241 ^c^
geosmin	13.1 ^b^	28.9 ^a^	8.90 ^b^	9.75 ^b^
heptanal	5.90 ^b^	10.0 ^a^	4.78 ^b^	6.61 ^b^
heptane	278 ^b^	341 ^a^	214 ^b^	208 ^b^
hexanal	13.2 ^b^	8.63 ^a^	8.04 ^b^	6.86 ^b^
hexanoic acid	1.79 ^ab^	1.78 ^a^	1.41 ^ab^	1.19 ^b^
hydrogen sulfide	2.43 ^a^	2.03 ^a^	2.20 ^a^	1.28 ^a^
indole	1.52 ^b^	1.84 ^b^	1.84 ^b^	0.94 ^a^
isobutyl alcohol	10,371 ^b^	14,787 ^a^	7508 ^b^	8890 ^b^
methyl mercaptan	11,842 ^b^	15,759 ^a^	8919 ^c^	10,019 ^bc^
nonanal	15.0 ^b^	45.6 ^a^	10.6 ^b^	23.7 ^b^
oct-2-en-1-ol	379 ^b^	878 ^a^	182 ^b^	293 ^b^
octanal	5.30 ^a^	6.31 ^a^	4.08 ^b^	4.87 ^b^
octane	54.1 ^b^	71.2 ^a^	43.7 ^b^	38.5 ^b^
pentanal	9.10 ^a^	7.25 ^bc^	11.0 ^ab^	6.36 ^c^
pentane	4681 ^ab^	5251 ^a^	4645 ^bc^	3672 ^c^
propanal	42.9 ^a^	13.7 ^b^	25.9 ^ab^	13.1 ^b^
trans-2-undecenal	9.53 ^b^	14.7 ^a^	7.74 ^b^	7.90 ^b^
trimethylamine	36.5 ^a^	30.5 ^ab^	27.0 ^b^	22.5 ^b^
trimethylpyrazine	16.3 ^b^	37.7 ^a^	9.26 ^b^	15.7 ^b^

**Table A3:** Effect of sex on the volatile profile of Atlantic salmon. Different letters indicate significant difference within each volatile (*p* < 0.05).

Volatile	Female (202 Days)	Female (242 Days)	Male (202 Days)	Male (242 Days)
(E,Z)-2,6-nonadienal	193 ^b^	252 ^a^	277 ^ab^	253 ^ab^
(E)-2-pentenal	96.2 ^b^	118 ^a^	126 ^ab^	119 ^ab^
1-hexanol	2.14 ^b^	3.34 ^a^	2.71 ^b^	3.65 ^a^
1-octanol	1.92 ^b^	2.65 ^a^	2.59 ^ab^	2.08 ^b^
1-octen-3-ol	369 ^b^	620 ^a^	629 ^ab^	552 ^b^
1-octen-3-one	0.78 ^a^	0.73 ^a^	0.78 ^a^	0.67 ^a^
1-pentanol	7.20 ^b^	9.95 ^a^	8.18 ^b^	9.56 ^ab^
1-penten-3-ol	9.08 ^a^	11.2 ^a^	10.4 ^a^	11.0 ^a^
2-butenal	1.35 ^c^	1.93 ^a^	1.62 ^bc^	1.87 ^ab^
2-decanone	0.81 ^b^	0.86 ^b^	1.47 ^a^	1.08 ^ab^
2-decenal	1.83 ^b^	2.31 ^ab^	2.97 ^a^	2.35 ^ab^
2-heptanone	5.10 ^a^	5.43 ^a^	5.66 ^a^	5.16 ^a^
2-heptenal	8.92 ^c^	12.0 ^b^	12.0 ^a^	10.7 ^bc^
2-hexenal	8.92 ^a^	11.0 ^a^	11.3 ^a^	10.4 ^a^
2-methyl naphthalene	7.62 ^a^	8.64 ^a^	11.0 ^a^	8.52 ^a^
2-nonanone	0.90 ^b^	1.22 ^a^	1.33 ^a^	0.68 ^b^
2-nonenal	41.1 ^b^	67.7 ^a^	68.5 ^a^	59.5 ^b^
2-octenal	0.80 ^b^	1.16 ^a^	1.01 ^ab^	1.23 ^a^
2-octene	5.05 ^b^	7.70 ^a^	7.93 ^a^	7.50 ^ab^
2-pentanone	1.01 ^a^	1.13 ^a^	1.37 ^a^	1.16 ^a^
2-penten-1-ol	6.69 ^a^	8.24 ^a^	7.66 ^a^	8.10 ^a^
2-pentene	10.5 ^b^	14.7 ^a^	12.1 ^b^	14.1 ^ab^
2-undecanone	0.79 ^ab^	0.88 ^a^	0.75 ^ab^	0.57 ^b^
2,4-decadienal	0.53 ^a^	0.59 ^a^	0.65 ^a^	0.60 ^a^
2,4-heptadienal	25.3 ^ab^	20.7 ^b^	29.0 ^a^	21.2 ^b^
3-hexen-1-ol	2031 ^b^	2342 ^a^	2570 ^ab^	2376 ^ab^
3-hexenal	3.50 ^a^	3.96 ^a^	3.71 ^a^	3.65 ^a^
decanal	1.43 ^c^	1.90 ^ab^	1.98 ^a^	1.48 ^bc^
heptanal	4.35 ^ab^	3.91 ^ab^	5.05 ^a^	3.84 ^b^
heptane	186 ^c^	344 ^a^	279 ^b^	290 ^b^
hexanal	5.53 ^ab^	5.35 ^ab^	6.74 ^a^	5.26 ^b^
hexanoic acid	0.96 ^a^	1.12 ^a^	1.05 ^a^	0.95 ^a^
nonanal	12.5 ^b^	16.4 ^ab^	17.6 ^a^	14.1 ^b^
oct-2-en-1-ol	318 ^b^	535 ^ab^	542 ^a^	476 ^b^
octanal	2.06 ^a^	2.49 ^a^	2.57 ^a^	2.35 ^a^
octane	37.3 ^c^	46.2 ^ab^	47.6 ^a^	42.6 ^bc^
pentanal	4.62 ^b^	6.82 ^a^	4.80 ^b^	5.94 ^a^
pentane	2487 ^b^	3497 ^a^	2924 ^b^	3286 ^ab^
propanal	17.0 ^ab^	13.9 ^b^	19.5 ^a^	14.3 ^b^
trans-2-undecenal	6.10 ^b^	8.58 ^a^	8.27 ^b^	7.52 ^b^
2-isopropyl-3-methoxypyrazine	5.21 ^b^	7.69 ^a^	9.22 ^a^	6.40 ^b^
2-methylisoborneol	2.11 ^a^	2.79 ^a^	3.03 ^a^	2.58 ^a^
2,3-butanediol	42.9 ^b^	67.0 ^a^	50.8 ^b^	60.4 ^ab^
alpha-terpinene	1.70 ^a^	1.68 ^a^	1.43 ^a^	1.75 ^a^
beta-caryophyllene	0.39 ^a^	0.74 ^a^	1.13 ^a^	0.88 ^a^
ethyl acetate	4.69 ^a^	4.82 ^a^	4.85 ^a^	4.65 ^a^
geosmin	10.1 ^b^	16.2 ^a^	16.6 ^a^	14.1 ^b^
isobutyl alcohol	8593 ^b^	9927 ^ab^	11,106 ^a^	10,039 ^ab^
3-methyl-1-butanol	16.0 ^b^	21.0 ^a^	22.8 ^a^	20.5 ^ab^
acetic acid	7.53 ^a^	11.6 ^a^	8.79 ^a^	9.25 ^a^
acetoin	4.22 ^a^	4.34 ^a^	4.37 ^a^	4.18 ^a^
acetone	9.79 ^c^	14.9 ^a^	13.2 ^b^	14.4 ^ab^
carbon disulfide	1043 ^b^	1314 ^a^	1523 ^a^	1345 ^ab^
dimethyl disulfide	5070 ^b^	6330 ^ab^	7238 ^a^	6341 ^ab^
dimethyl sulfide	2.85 ^c^	4.79 ^a^	3.27 ^c^	4.42 ^b^
formaldehyde	1215 ^a^	1267 ^a^	1411 ^a^	1304 ^a^
hydrogen sulfide	0.65 ^a^	0.76 ^a^	0.71 ^a^	0.72 ^a^
indole	0.94 ^a^	0.84 ^a^	0.73 ^a^	1.06 ^a^
methyl mercaptan	9534 ^a^	10,613 ^a^	12,023 ^a^	10,825 ^a^
trimethylamine	20.9 ^a^	25.3 ^a^	26.2 ^a^	23.1 ^a^
ammonia	487 ^c^	711 ^a^	562 ^bc^	672 ^b^
dimethylamine	243 ^b^	393 ^a^	376 ^bc^	360 ^b^
2-acetyl furan	1.19 ^a^	1.39 ^a^	1.58 ^a^	1.60 ^a^
2-ethyl-2,5, dimethylpyrazine	1.84 ^b^	3.06 ^a^	3.14 ^ab^	2.06 ^b^
2-ethylfuran	18.7 ^b^	23.9 ^a^	26.6 ^ab^	23.9 ^ab^
trimethylpyrazine	14.8 ^b^	20.7 ^ab^	22.9 ^a^	19.8 ^b^

**Table A4:** Effect of the presence of skin on the volatile profile of Atlantic salmon. Different letters indicate significant difference within each volatile (*p* < 0.05).

Volatile	Fillet Without Skin (202 Days)	Fillet Without Skin (242 Days)	Fillet with Skin (202 Days)	Fillet with Skin (242 Days)
(E,Z)-2,6-nonadienal	199 ^b^	209 ^b^	272 ^a^	295 ^a^
(E)-2-pentenal	99.4 ^c^	103 ^bc^	123 ^ab^	133 ^a^
1-hexanol	2.17 ^c^	3.15 ^b^	2.68 ^bc^	3.84 ^a^
1-octanol	1.86 ^b^	1.88 ^b^	2.66 ^a^	2.86 ^a^
1-octen-3-ol	373 ^c^	390 ^c^	624 ^b^	782 ^a^
1-octen-3-one	0.82 ^a^	0.62 ^a^	0.75 ^a^	0.77 ^a^
1-pentanol	6.68 ^b^	8.70 ^b^	8.70 ^b^	10.81 ^a^
1-penten-3-ol	7.63 ^b^	11.0 ^a^	11.9 ^a^	11.1 ^a^
2-butenal	1.24 ^c^	1.76 ^ab^	1.72 ^b^	2.04 ^a^
2-decanone	1.26 ^a^	0.93 ^a^	1.02 ^a^	1.01 ^a^
2-decenal	2.35 ^a^	2.21 ^a^	2.45 ^a^	2.45 ^a^
2-heptanone	5.08 ^ab^	4.63 ^b^	5.68 ^ab^	5.96 ^a^
2-heptenal	8.44 ^c^	8.65 ^c^	12.5 ^b^	13.9 ^a^
2-hexenal	8.27 ^c^	8.74 ^bc^	12.0 ^ab^	12.7 ^b^
2-methyl naphthalene	5.95 ^b^	5.48 ^b^	12.7 ^a^	11.7 ^a^
2-nonanone	0.99 ^ab^	0.83 ^b^	1.23 ^ab^	1.08 ^a^
2-nonenal	40.2 ^c^	40.5 ^c^	69.4 ^b^	86.8 ^a^
2-octenal	0.77 ^b^	1.07 ^b^	1.04 ^ab^	1.31 ^a^
2-octene	4.92 ^c^	6.39 ^bc^	8.06 ^ab^	8.81 ^a^
2-pentanone	1.14 ^a^	1.26 ^a^	1.25 ^a^	1.03 ^a^
2-penten-1-ol	5.62 ^b^	8.13 ^a^	8.73 ^a^	8.21 ^a^
2-pentene	9.87 ^b^	12.9 ^b^	12.9 ^ab^	15.99 ^a^
2-undecanone	0.54 ^c^	0.66 ^bc^	1.00 ^a^	0.79 ^ab^
2,4-decadienal	0.62 ^ab^	0.50 ^b^	0.56 ^ab^	0.69 ^a^
2,4-heptadienal	25.4 ^ab^	21.5 ^b^	28.8 ^a^	20.1 ^b^
3-hexen-1-ol	2087 ^b^	2084 ^b^	2514 ^a^	2634 ^a^
3-hexenal	3.55 ^a^	3.30 ^a^	3.66 ^a^	4.31 ^a^
decanal	1.40 ^b^	1.32 ^b^	2.01 ^a^	2.05 ^a^
heptanal	4.29 ^b^	3.05 ^c^	5.11 ^a^	4.69 ^ab^
heptane	216 ^b^	249 ^b^	249 ^b^	385 ^a^
hexanal	5.50 ^b^	5.13 ^b^	6.77 ^a^	5.47 ^b^
hexanoic acid	1.05 ^ab^	0.86 ^b^	0.96 ^b^	1.21 ^a^
nonanal	10.2 ^b^	10.2 ^b^	19.9 ^a^	20.3 ^a^
oct-2-en-1-ol	322 ^c^	337 ^c^	538 ^b^	674 ^a^
octanal	1.96 ^b^	2.22 ^ab^	2.67 ^a^	2.63 ^a^
octane	35.0 ^c^	33.6 ^c^	49.9 ^b^	55.2 ^a^
pentanal	3.80 ^c^	7.93 ^a^	5.62 ^b^	4.83 ^b^
pentane	2235 ^b^	3379 ^a^	3177 ^a^	3405 ^a^
propanal	17.1 ^ab^	14.4 ^b^	19.4 ^a^	13.7 ^b^
trans-2-undecenal	5.98 ^c^	6.63 ^bc^	8.39 ^b^	9.48 ^a^
2-isopropyl-3-methoxypyrazine	6.18 ^b^	5.92 ^b^	8.25 ^a^	8.18 ^a^
2-methylisoborneol	1.76 ^c^	1.96 ^bc^	3.39 ^ab^	3.41 ^a^
2,3-butanediol	39.5 ^b^	69.1 ^a^	54.2 ^ab^	58.3 ^a^
alpha-terpinene	1.46 ^a^	1.76 ^a^	1.66 ^a^	1.67 ^a^
beta-caryophyllene	0.60 ^a^	0.70 ^a^	0.92 ^a^	0.91 ^a^
ethyl acetate	4.40 ^a^	4.70 ^a^	5.14 ^a^	4.77 ^a^
geosmin	11.1 ^c^	11.3 ^bc^	15.5 ^b^	19.0 ^a^
isobutyl alcohol	8486 ^b^	8467 ^b^	11,213 ^a^	11,499 ^a^
3-methyl-1-butanol	17.7 ^a^	20.2 ^a^	21.2 ^a^	21.3 ^a^
acetic acid	6.84 ^a^	11.0 ^a^	9.49 ^a^	9.89 ^a^
acetoin	3.96 ^a^	4.23 ^a^	4.63 ^a^	4.30 ^a^
acetone	10.1 ^c^	13.8 ^b^	12.9 ^b^	15.6 ^a^
carbon disulfide	1016 ^b^	1016 ^b^	1550 ^a^	1642 ^a^
dimethyl disulfide	5011 ^b^	4982 ^b^	7297 ^a^	7688 ^a^
dimethyl sulfide	2.62 ^d^	4.26 ^b^	3.50 ^c^	4.95 ^a^
formaldehyde	1201 ^b^	1171 ^b^	1425 ^a^	1400 ^a^
hydrogen sulfide	0.68 ^a^	0.60 ^a^	0.68 ^a^	0.88 ^a^
indole	0.58 ^a^	0.94 ^a^	1.09 ^a^	0.95 ^a^
methyl mercaptan	9404 ^b^	9264 ^b^	12,153 ^a^	12,174 ^a^
trimethylamine	22.8 ^a^	23.7 ^a^	24.3 ^a^	24.7 ^a^
ammonia	443 ^c^	527 ^bc^	605 ^b^	856 ^a^
dimethylamine	249 ^c^	309 ^bc^	371 ^b^	444 ^a^
2-acetyl furan	1.31 ^a^	1.40 ^a^	1.46 ^a^	1.58 ^a^
2-ethyl-2,5, -dimethylpyrazine	2.00 ^b^	2.11 ^b^	2.98 ^ab^	3.01 ^a^
2-ethylfuran	18.1 ^b^	19.1 ^b^	27.3 ^a^	28.7 ^a^
trimethylpyrazine	14.3 ^b^	14.5 ^b^	23.3 ^a^	25.9 ^a^

## References

[1] Jørgensen L.V., Huss H.H., Dalgaard P.J. (2001). Significance of Volatile Compounds Produced by Spoilage Bacteria in Vacuum-Packed Cold-Smoked Salmon (*Salmo salar*) Analyzed by GC-MS and Multivariate Regression. Agric. Food Chem..

[2] Jónsdóttir R., Ólafsdóttir G., Chanie E., Haugen J.-E. (2008). Volatile compounds suitable for rapid detection as quality indicators of cold smoked salmon (*Salmo salar*). Food Chem..

[3] Luo J., Frank D., Arcot J. (2024). Creating alternative seafood flavour from non-animal ingredients: A review of key flavour molecules relevant to seafood. Food Chem. X.

[4] Amaral A.B., Silva M.V., Lannes S.C.S. (2018). Lipid oxidation in meat: Mechanisms and protective factors—A review. Food Sci. Technol. (Campinas).

[5] Fontes P.R., Gomide L.A.M., Romas E.M., Stringheta P.C. (2005). Color evaluation of carbon monoxide treated porcine blood. Meat Sci..

[6] Conz A., Davoli E., Franchi C., Diomede L. (2021). Seafood loss prevention and waste reduction. Food Qual. Saf..

[7] FAO (2020). The State of World Fisheries and Aquaculture 2020: Sustainability in Action.

[8] Undercurrent News Norway Led US Atlantic Salmon Import Rebound in Q1. https://www.undercurrentnews.com/2025/05/26/norway-led-us-atlantic-salmon-import-rebound-in-q1/.

[9] Yu Y.J., Yang S.P., Lin T., Qian Y.F., Xie J., Hu C. (2020). Effect of Cold Chain Logistic Interruptions on Lipid Oxidation and Volatile Organic Compounds of Salmon (*Salmo salar*) and Their Correlations With Water Dynamics. Front. Nutr..

[10] Bendiksen E., Jobling M. (2003). Effects of temperature and feed composition on essential fatty acid (n-3 and n-6) retention in Atlantic salmon (*Salmo salar* L.) parr. Fish Physiol. Biochem..

[11] Handeland S.O., Imsland A.K., Björnsson B.T., Stefansson S.O. (2013). Long-term effects of photoperiod, temperature and their interaction on growth, gill Na+, K+-ATPase activity, seawater tolerance and plasma growth-hormone levels in Atlantic salmon *Salmo salar*. J. Fish Biol..

[12] Pino-Martínez E., Balseiro P., Pedrosa C., Haugen T.S., Fleming M.S., Handeland S.O. (2021). The effect of photoperiod manipulation on Atlantic salmon growth, smoltification and sexual maturation: A case study of a commercial RAS. Aquac. Res..

[13] Clercin N., Druschel G. (2019). Influence of Environmental Factors on the Production of MIB and Geosmin Metabolites by Bacteria in a Eutrophic Reservoir. Water Resour. Res..

[14] Suurnäkki S., Gómez-Saez G.V., Rantala-Ylinen A., Jokela J., Fewer D.P., Sivonen K. (2015). Identification of geosmin and 2-methylisoborneol in cyanobacteria and molecular detection methods for the producers of these compounds. Water Res..

[15] Parlapani F.F., Anagnostopoulos D.A., Karamani E., Mallouchos A., Haroutounian S.A., Boziaris I.S. (2023). Growth and Volatile Organic Compound Production of Pseudomonas Fish Spoiler Strains on Fish Juice Agar Model Substrate at Different Temperatures. Microorganisms.

[16] Doyle M.E. (2007). Microbial Food Spoilage—Losses and Control Strategies.

[17] Zhao S., Yu J., Xi L., Kong X., Pei J., Jiang P., Gao R., Jin W. (2024). Sex-Specific Lipid Profiles and Flavor Volatiles in Giant Salamander (*Andrias davidianus*) Tails Revealed by Lipidomics and GC-IMS. Foods.

[18] Chaliha M., Cusack A., Currie M., Sultanbawa Y., Smyth H. (2013). Effect of Packaging Materials and Storage on Major Volatile Compounds in Three Australian Native Herbs. J. Agric. Food Chem..

[19] Sendón García R., Sanches Silva A., Cooper I., Franz R., Paseiro Losada P. (2006). Revision of analytical strategies to evaluate different migrants from food packaging materials. Trends Food Sci. Technol..

[20] Gram L., Huss H.H. (1996). Microbiological spoilage of fish and fish products. Int. J. Food Microbiol..

[21] Maqsood S., Benjakul S., Shahidi F. (2013). Emerging Role of Phenolic Compounds as Natural Food Additives in Fish and Fish Products. Crit. Rev. Food Sci. Nutr..

[22] Sae-leaw T., Benjakul S. (2014). Fatty acid composition, lipid oxidation, and fishy odour development in seabass (*Lates calcarifer*) skin during iced storage. Eur. J. Lipid Sci. Technol..

[23] Lindholm-Lehto P.C., Vielma J. (2019). Controlling of geosmin and 2-methylisoborneol induced off-flavours in recirculating aquaculture system farmed fish—A review. Aquac. Res..

[24] Pino Martinez E., Balseiro P., Fleming M.S., Stefansson S.O., Norberg B., Imsland A.K.D., Handeland S.O. (2023). Interaction of Temperature and Photoperiod on Male Postsmolt Maturation of Atlantic Salmon (*Salmo salar* L.). Aquaculture.

[25] Venkateshwarlu G., Let M.B., Meyer A.S., Jacobsen C. (2004). Chemical and Olfactometric Characterization of Volatile Flavor Compounds in a Fish Oil Enriched Milk Emulsion. J. Agric. Food Chem..

[26] Piveteau F., Guen S., Gandemer G., Baud J., Prost C., Demaimay M. (2000). Aroma of Fresh Oysters Crassostrea gigas: Composition and Aroma Notes. J. Agric. Food Chem..

[27] Ross C., Smith D. (2006). Use of Volatiles as Indicators of Lipid Oxidation in Muscle Foods. Compr. Rev. Food Sci. Food Saf..

[28] Elliott J.M., Hurley M.A. (1997). A functional model for maximum growth of Atlantic Salmon parr, *Salmo salar*, from two populations in northwest England. Funct. Ecol..

[29] Bernthal F.R., Seaman B.W., Rush E., Armstrong J.D., McLennan D., Nislow K.H., Metcalfe N.B. (2023). High summer temperatures are associated with poorer performance of underyearling Atlantic salmon (*Salmo salar*) in upland streams. J. Fish Biol..

[30] Forseth T., Hurley M., Jensen A., Elliott J. (2001). Functional models for growth and food consumption of Atlantic salmon parr, *Salmo salar*, from a Norwegian river. Freshw. Biol..

[31] Feidantsis K., Pörtner H., Antonopoulou E., Michaelidis B. (2014). Synergistic effects of acute warming and low pH on cellular stress responses of the gilthead seabream *Sparus aurata*. J. Comp. Physiol. B.

[32] Mittakos I., Nathanailides C.I., Kokokiris L.E., Barbouti A., Bitchava K., Gouva E., Kolygas M.N., Terzidis M.A., Kontominas M.G. (2025). Antioxidant Capacity, Lipid Oxidation, and Quality Traits of Slow- and Fast-Growing Meagre (*Argyrosomus regius*) Fillets During Cold Storage. Antioxidants.

[33] Osako K., Kuwahara K., Nozaki Y. (2003). Seasonal variations in gel-forming ability of rabbit fish. Fish. Sci..

[34] Yin P., Björnsson B.T., Fjelldal P.G., Saito T., Remø S.C., Edvardsen R.B., Hansen T., Sharma S., Olsen R.E., Hamre K. (2022). Impact of Antioxidant Feed and Growth Manipulation on the Redox Regulation of Atlantic Salmon Smolts. Antioxidants.

[35] Nemova N.N., Nefedova Z.A., Pekkoeva S.N., Voronin V.P., Shulgina N.S., Churova M.V., Murzina S.A. (2020). The Effect of the Photoperiod on the Fatty Acid Profile and Weight in Hatchery-Reared Underyearlings and Yearlings of Atlantic Salmon *Salmo salar* L.. Biomolecules.

[36] Yin P., Saito T., Fjelldal P.G., Björnsson B.T., Remø S.C., Hansen T.J., Sharma S., Olsen R.E., Hamre K. (2023). Seasonal Changes in Photoperiod: Effects on Growth and Redox Signaling Patterns in Atlantic Salmon Postsmolts. Antioxidants.

[37] Heise K., Puntarulo S., Nikinmaa M., Abele D., Pörtner H. (2006). Oxidative stress during stressful heat exposure and recovery in the North Sea eelpout *Zoarces viviparus* L.. J. Exp. Biol..

[38] Olsvik P., Vikeså V., Lie K., Hevrøy E. (2013). Transcriptional responses to temperature and low oxygen stress in Atlantic salmon studied with next-generation sequencing technology. BMC Genom..

[39] Landry J.D., Blanch E.W., Torley P.J. (2023). Chemical Indicators of Atlantic Salmon Quality. Food Rev. Int..

[40] Kaur M., Atif F., Ali M., Rehman H., Raisuddin S.J. (2005). Heat stress-induced alterations of antioxidants in the freshwater fish Channa punctata Bloch. Fish Biol..

[41] Dalsvåg H., Cropotova J., Jambrak A., Janči T., Španěl P., Dryahina K., Rustad T. (2021). Mass Spectrometric Quantification of Volatile Compounds Released by Fresh Atlantic Salmon Stored at 4 °C under Modified Atmosphere Packaging and Vacuum Packaging for up to 16 Days. ACS Food Sci. Technol..

[42] Ólafsdóttir G., Jónsdóttir R., Lauzon H.L., Luten J.B., Kristbergsson K. (2005). Characterization of Volatile Compounds in Chilled Cod (*Gadus morhua*) Fillets by Gas Chromatography and Detection of Quality Indicators by an Electronic Nose. J. Agric. Food Chem..

[43] Lindholm-Lehto P.C., Koskela J., Kaseva J., Vielma J. (2020). Accumulation of Geosmin and 2-methylisoborneol in European Whitefish *Coregonus lavaretus* and Rainbow Trout *Oncorhynchus mykiss* in RAS. Fishes.

[44] Gómez-Boronat M., Sáiz N., Delgado M., Pedro N., Isorna E. (2018). Time-Lag in Feeding Schedule Acts as a Stressor That Alters Circadian Oscillators in Goldfish. Front. Physiol..

[45] Imsland A.K.D., Roth B., Døskeland I., Fjelldal P.G., Stefansson S.O., Handeland S., Mikalsen B. (2019). Flesh quality of Atlantic salmon smolts reared at different temperatures and photoperiods. Aquac. Res..

[46] Sáiz N., Gómez-Boronat M., Pedro N., Delgado M., Isorna E. (2021). The Lack of Light-Dark and Feeding-Fasting Cycles Alters Temporal Events in the Goldfish (*Carassius auratus*) Stress Axis. Animals.

[47] Doan H., Yamaka S., Pornsopin P., Jaturasitha S., Faggio C. (2020). Proximate and Nutritional Content of Rainbow Trout (*Oncorhynchus mykiss*) Flesh Cultured in a Tropical Highland Area. Braz. Arch. Biol. Technol..

[48] Tang H., Chen L., Xiao C., Wu T.J. (2009). Fatty acid profiles of muscle from large yellow croaker (*Pseudosciaena crocea* R.) of different age. Zhejiang Univ. Sci. B.

[49] Stansby M.E. (1954). Composition of certain species of fresh-water fish. I. Introduction: The determination of the variation of composition of fish. J. Food Sci..

[50] Jacquot R. (1961). CHAPTER 6–Organic Constituents of Fish and Other Aquatic Animal Foods. Fish as Food.

[51] Matulić D., Blažina M., Pritišanac E., Čolak S., Bavčević L., Barić R., Križanac S., Vitlov B., Šuran J., Perović I.S. (2024). Growth, Fatty Acid Profile and Malondialdehyde Concentration of Meagre Argyrosomus regius Fed Diets with Different Lipid Content. Appl. Sci..

[52] Kunyaboon S., Thumanu K., Park J.W., Khongla C., Yongsawatdigul J. (2021). Evaluation of Lipid Oxidation, Volatile Compounds and Vibrational Spectroscopy of Silver Carp (*Hypophthalmichthys molitrix*) during Ice Storage as Related to the Quality of Its Washed Mince. Foods.

[53] Ma R., Meng Y., Zhang W., Mai K. (2019). Comparative study on the organoleptic quality of wild and farmed large yellow croaker *Larimichthys crocea*. J. Oceanol. Limnol..

[54] Sarika A.R., Lipton A.P., Aishwarya M.S. (2019). Biopreservative Efficacy of Bacteriocin GP1 of Lactobacillus rhamnosus GP1 on Stored Fish Filets. Front. Nutr..

[55] Kuswandi B., Hasanah F., Pratoko D.K., Kristiningrum N. (2022). Colorimetric Paper-Based Dual Indicator Label for Real-Time Monitoring of Fish Freshness. Food Technol. Biotechnol..

[56] Bower C., Malemute C., Bechtel P. (2011). ENDOGENOUS PROTEASE ACTIVITY IN BY-PRODUCTS OF PINK SALMON (*ONCORHYNCHUS GORBUSCHA*). J. Food Biochem..

[57] Duflos G., Coin V.M., Cornu M., Antinelli J.F., Malle P. (2005). Determination of volatile compounds to characterize fish spoilage using headspace/mass spectrometry and solid-phase microextraction/gas chromatography/mass spectrometry. J. Sci. Food Agric..

[58] Schrader K., Summerfelt S.T. (2010). Distribution of Off-Flavor Compounds and Isolation of Geosmin-Producing Bacteria in a Series of Water Recirculating Systems for Rainbow Trout Culture. N. Am. J. Aquac..

[59] Tucker C.S. (2000). Off-Flavor Problems in Aquaculture. Rev. Fish. Sci..

[60] Percival S., Drabsch P., Glencross B. (2008). Determining factors affecting muddy-flavour taint in farmed barramundi, Lates calcarifer. Aquaculture.

[61] Howgate P. (2004). Tainting of farmed fish by geosmin and 2-methyl-iso-borneol: A review of sensory aspects and of uptake/depuration. Aquaculture.

[62] Shahidi F., Zhong Y. (2008). Lipid oxidation and improving the oxidative stability. Chem. Soc. Rev..

[63] House A.H., Debes P.V., Kurko J., Erkinaro J., Käkelä R., Primmer C.R. (2020). Sex-specific lipid profiles in the muscle of Atlantic salmon juveniles. Biorxiv.

[64] Jin W., Zhao S., Li J., Cheng K., Xi L., Pei J., Gao R., Jiang P. (2024). Unraveling sex-specific lipids and flavor volatiles in giant salamander (*Andrias davidianus*) livers via lipidomics and GC-IMS. Food Chem. X.

[65] Martin N.B., Houlihan D.F., Talbot C., Palmer R.M. (1993). Protein metabolism during sexual maturation in female Atlantic salmon (*Salmo salar* L.). Fish Physiol. Biochem..

[66] Wang Q., Lu S., Tao Y., Hua J., Zhuge Y., Chen W., Qiang J. (2024). Characteristic Muscle Quality Parameters of Male Largemouth Bass (Micropterus salmoides) Distinguished from Female and Physiological Variations Revealed by Transcriptome Profiling. Biology.

[67] Dhurmeea Z., Pethybridge H., Appadoo C., Bodin N. (2018). Lipid and fatty acid dynamics in mature female albacore tuna (*Thunnus alalunga*) in the western Indian Ocean. PLoS ONE.

[68] Karbsri W., Hamzeh A., Yongsawatdigul J. (2024). Changes in volatile compounds and lipid oxidation in various tissues of Nile tilapia (*Oreochromis niloticus*) during ice storage. J. Food Sci..

[69] Ackman R., Heras H., Zhou S. (1996). SALMON LIPID STORAGE SITES AND THEIR ROLE IN CONTAMINATION WITH WATER-SOLUBLE PETROLEUM MATERIALS. J. Food Lipids.

[70] Aursand M., Bleivik B., Rainuzzo J., Jørgensen L., Mohr V. (1994). Lipid distribution and composition of commercially farmed atlantic salmon (*salmosalar*). J. Sci. Food Agric..

[71] Głowacz-Różyńska A., Tynek M., Malinowska-Pańczyk E., Martysiak-Żurowska D., Pawłowicz R., Kołodziejska I. (2016). Comparison of oil yield and quality obtained by different extraction procedures from salmon (*Salmo salar*) processing byproducts. Eur. J. Lipid Sci. Technol..

[72] Wójciak K., Dolatowski Z., Kołożyn-Krajewska D., Trząskowska M. (2012). Comparison of oil yield and quality obtained by different extraction procedures from salmon (*Salmo salar*) processing byproducts. J. Food Qual..

[73] Zhang C., Zhu S., Wu H., Jatt A., Pan Y., Zeng M. (2016). Quorum Sensing Involved in the Spoilage Process of the Skin and Flesh of Vacuum-Packaged Farmed Turbot (*Scophthalmus maximus*) Stored at 4 °C. J. Food Sci..

[74] Jyothylakshmi K., Nandakumar S., Kumar M.G.S. (2020). Bacterial pollution indicators associated in the tissues of an estuarine fish mugil cephalusfrom Ashtamudi lake, aRAMSAR site(Kerala, India). Sustain. Agri. Food Environ. Res..

[75] Webster T., Consuegra S., Hitchings M., Leániz C. (2018). Inter-population variation in the Atlantic salmon microbiome reflects environmental and genetic diversity. Biorxiv.

[76] Ellison A., Wilcockson D., Cable J. (2021). Circadian dynamics of the teleost skin immune-microbiome interface. Biorxiv.

[77] Cheng H., Wang J., Xie J. (2023). Progress on odor deterioration of aquatic products: Characteristic volatile compounds, analysis methods, and formation mechanisms. Food Biosci..

[78] Ganguly S., Mahanty A., Mitra T., Raman R.K., Mohanty B.P. (2017). Volatile compounds in hilsa (*Tenualosa ilisha, Hamilton*) as detected by static headspace gas chromatography and mass spectrometry. J. Food Process. Preserv..

[79] Natale C.D., Ólafsdóttir G. (2009). Electronic Nose and Electronic Tongue. Fish. Prod..

[80] Di Lucia F., Lacivita V., Nobile M.A.D., Conte A. (2021). Improving the Storability of Cod Fish-Burgers According to the Zero-Waste Approach. Foods.

[81] Oveland E., Bøkevoll A., Araujo P., Hemre G. (2024). Frozen storage procedures for salmon and plaice samples: Nutrient composition and implications for preservation. J. Food Sci..

[82] Nguyen M.V., Phan L.M.T. (2018). Influences of Bleeding Conditions on the Quality and Lipid Degradation of Cobia (*Rachycentron canadum*) Fillets During Frozen Storage. Turk. J. Fish. Aquat. Sci..

[83] Subbaiah K., Majumdar R.K., Choudhury J., Priyadarshini B.M., Dhar B., Roy D., Saha A., Maurya P. (2015). Protein Degradation and Instrumental Textural Changes in Fresh Nile Tilapia (*Oreochromis niloticus*) during Frozen Storage. J. Food Process. Preserv..

